# Perfusion Index Predicts the Effectiveness of Supraclavicular Brachial Plexus Block in Children Under General Anesthesia: A Randomized Controlled Trial

**DOI:** 10.1155/anrp/5583145

**Published:** 2025-02-22

**Authors:** Tiantian Chu, Siqi Zhou, Ting Peng, Hong Tao, Han Chen, Xu Yan, Yueyang Xin, Zhang Tian, Jinxu Wang, Lingli Deng, Aijun Xu

**Affiliations:** ^1^Department of Anaesthesiology, Hubei Key Laboratory of Geriatric Anaesthesia and Perioperative Brain Health, and Wuhan Clinical Research Center for Geriatric Anaesthesia, Tongji Hospital, Tongji Medical College, Huazhong University of Science and Technology, Wuhan 430030, China; ^2^Department of Anesthesiology, Guizhou Provincial People's Hospital, Guiyang, Guizhou 550002, China; ^3^Department of Anaesthesiology, Beijing Chao-Yang Hospital, Capital Medical University, Beijing 100020, China

## Abstract

**Objectives:** This study aimed to assess the predictive value of perfusion index (PI) in determining the effectiveness of supraclavicular block (SCB) in children under sevoflurane or propofol general anesthesia.

**Methods:** In this randomized controlled study, 104 children who underwent elective upper extremity surgery under sevoflurane or propofol anesthesia were scheduled to be enrolled. The primary outcome was the effects of PI in predicting the effectiveness of SCB under general anesthesia. The PI value was obtained through pulse oximetries. Secondary outcomes include hemodynamic data, supplementary opioid doses, agitation score, pain score, and postoperative complications.

**Results:** A total of 103 pediatric patients were analyzed. PI increased rapidly after anesthesia induction, and there was no significant difference in PI in the blocked side between the propofol group (PRO group) and sevoflurane group (SEV group). At 10 min after the block, PI in the blocked side was higher than that in the unblocked side in both groups (*p* < 0.05). However, PI showed low sensitivity and specificity in predicting the effect of SCB at 10 min in both groups. At PACU, PI exhibited a high sensitivity (0.837 vs. 0.796) and specificity (0.721 vs. 0.898) for SCB at cutoff values of 5.91 and 6.67 in both PRO and SEV groups. The area under the receiver operating characteristic curve (AUROC) values were 0.834 (95% CI 0.750–0.918) and 0.895 (95% CI 0.832–0.959).

**Conclusion:** PI demonstrates limited sensitivity and specificity in predicting the effect of SCB at 10 min after block under general anesthesia. However, PI may serve as a more appropriate indicator to guide the necessity for supplemental analgesia in PACU.

**Trial Registration:** ClinicalTrials.gov identifier: NCT04216823

## 1. Introduction

Supraclavicular block (SCB) is the most common type of brachial plexus block and provides analgesia for upper extremity operations. It has become increasingly popular in children because it provides prolonged and high-quality analgesia, which not only reduces the adverse effect of general anesthetics on neurodevelopment but also improves the healthcare experience of patients [1, 2]. Although pediatric SCB needs to be performed under sedation or general anesthesia (GA), it is safe and even has a lower risk of complications compared with awake block [3, 4]. However, the traditional approach to evaluating the success of the block is based on sensory and motor blocks, which is not suitable for pediatric patients under GA [5].

The peripheral perfusion index (PI), calculated as the ratio of pulsatile and nonpulsatile blood flows, reflects peripheral perfusion and is mainly affected by sympathetic tone [6]. After a successful SCB, sympathetic nerve tone decreases and peripheral vasodilation results in an increased local perfusion [5]. Therefore, most studies have demonstrated that a rapid increase in PI is a reliable predictor of successful SCB [7–9]. However, current research mainly focuses on awake adults, with less research in children. Still, there is a lack of relevant research on PI used for children under GA.

Propofol and sevoflurane are commonly used for the induction and maintenance of GA in children, but they both have vasodilation. Ryu et al. have shown that PI changes differently under different anesthetic agents [10]. However, no studies have reported the effect of propofol or sevoflurane anesthesia on the predictive performance of PI. Thus, we aimed to assess the effect of PI on predicting the effectiveness of SCB in children under propofol or sevoflurane anesthesia.

## 2. Methods

This is a prospective, single-center, randomized controlled study. The study was conducted at Tongji Hospital and adhered to the tenets of the Declaration of Helsinki. The trial was approved by the Ethics Committee of the Tongji Medical College of Huazhong University of Science and Technology, Wuhan, China, on July 03, 2020 (Chairperson Prof Hui Chen; ID: 2020S134). Written informed consent was obtained from all the parents or legally authorized representatives of pediatric patients.

### 2.1. Participants

The study included patients aged 1 month to 12 years with ASA physical status I-II who were scheduled for elective upper extremity surgery. Children with abnormal behavior (i.e., behavioral characteristics significantly deviate from normal developmental patterns, such as autism spectrum disorder, and emotional disturbance), infection at the puncture site, other regional anesthesia contraindications, or allergies to known ingredients of experimental drugs were excluded.

### 2.2. Randomization and Blinding

Patients were randomly assigned to either the propofol group (PRO group) or sevoflurane group (SEV group) based on a computer-generated randomization schedule in a 1:1 ratio. Randomized numbers were sealed in numbered opaque envelopes and would be opened by an investigator who was not involved in the study. The anesthesiologists were the only staff who were unblinded to group assignments, but they were not involved in the recording and analysis of the data.

### 2.3. Intervention

Intravenous access was established in the ward. All patients were administered 0.1 mg·kg^−1^ of midazolam intravenously 15 min before surgery in the preoperative waiting room. After children were transferred to the operating room, the electrocardiogram (ECG), blood pressure (BP), respiratory rate (RR), and pulse oxygen saturation (S_p_O_2_) were routinely monitored. Hundred percent oxygen at a rate of 6 L·min^−1^ was used for mask ventilation. 250 mL of normal saline was infused at a rate of 5 mL·kg^−1^·h^−1^ during the operation. Both groups received 0.2 μg·kg^−1^ of sufentanil, 0.01 mg·kg^−1^ of phencyclidine hydrochloride, and 0.1 mg·kg^−1^ of dexamethasone. Additionally, the PRO group injected 3 mg·kg^−1^ of propofol (Corden Pharma S.P.A., Italy) and the SEV group inhaled 5%–8% sevoflurane (Shanghai Hengrui Pharmaceutical Co., Ltd., China) for anesthesia induction. The laryngeal mask (LMA) was placed after achieving an adequate depth of anesthesia. Anesthesia was maintained with 3–10 mg·kg^−1^·h^−1^ of propofol or 1.0 MAC of sevoflurane. During surgery, 50% of oxygen (2 L·min^−1^) was supplied and the end-tidal carbon dioxide partial pressure (P_ET_CO_2_) was maintained at 35–45 mmHg.

SCB was performed under ultrasound guidance (6–13 MHz; SonoSite, Bothell, USA) by an experienced anesthesiologist. The patient's head was turned to the contralateral side, and the shoulder was padded 5–10 cm higher. A 50-mm, 22-gauge insulated nerve block needle (B.Braun, Germany) was inserted from lateral to medial toward the brachial plexus using an in-plane method. Hydrodissection with saline was utilized to confirm the needle tip's location and to separate the nerve cords from surrounding tissues. Subsequently, 0.4 mL·kg^−1^ of 0.25% ropivacaine (Guangdong Jiabo Pharmaceutical Co., Ltd., China) was injected to surround all the nerve cords [1, 11, 12]. Complete block was defined as no significant fluctuation in BP, heart rate (HR), or RR in response to incision stimulation during the operation, i.e., BP or HR increased no more than 10% from baseline and RR increased no more than 20%. When nonsingle nerves innervated the surgical site, there was a specific innervated area that caused BP, HR, and RR fluctuations, while other areas did not fluctuate significantly during surgery and were considered partial blocks. The standard for block failure was that surgery on any innervated area causes significant fluctuations in BP, HR, and RR. In case of partial block and block failure, a single dose of sufentanil (0.1 μg·kg^−1^) and a continuous infusion of remifentanil (0.1–0.2 μg·kg^−1^·min^−1^) were used for supplemental analgesia.

The operation was performed by a team of experienced surgeons and began 15 min after the block. Propofol and sevoflurane were discontinued 5 min before the end of the surgery. LMA was removed when RR > 8 bpm, tidal volume > 6 L/min, and protective reflex recovery [13]. Then, patients were transferred to the postanesthesia care unit (PACU) and monitored by a specific nurse. Emergence agitation (Watcha score), postoperative pain (the Children's and Infants' Postoperative Pain Scale [CHIPPS]), and complications were recorded during PACU [14, 15]. 1 mg·kg^−1^ of flurbiprofen axetil (maximum dose 50 mg) was slowly intravenously infused for routine postoperative analgesia. The infusion was initiated 8–10 h after block application on the day of surgery and administered once daily for the first and second postoperative days. If the CHIPPS score is > 4, 6.25 mg of diclofenac sodium suppository would be administered for additional analgesia.

### 2.4. Primary Outcome

The primary outcome was the effects of PI in predicting the effectiveness of SCB in children under GA. The PI value was obtained through pulse oximetries (Shenzhen Mindray Bio-Medical Electronics Co., Ltd., China) placed simultaneously on the bilateral index and little fingers. PI was recorded at the following time points: before the anesthesia induction (T0, baseline), immediately after LMA placement (T1), 0 min (T2), 5 min (T3), 10 min (T4), 15 min (T5) after SCB, immediately after surgery (T6), after removal of LMA (T7), and in PACU (T8).

### 2.5. Secondary Outcomes

The secondary outcome included the mean arterial pressure (MAP), HR, and PI ratio (baseline PI/PI at 10 min after block). Supplementary opioid doses, agitation score, pain score, and postoperative complications including nausea and vomiting, nerve damage, respiratory depression, etc., were also recorded.

### 2.6. Sample Size

MedCalc Software Version 14 was used to calculate the sample size. It was assumed that PI has a superior predictive effect, with an area under the receiver operating characteristic curve (AUROC) of 0.8 compared to the null hypothesis AUROC of 0.5. The preliminary estimated sample size was 47 cases, with *α* = 0.05 and *β* = 10%. Considering a dropout rate of 10% finally included 52 cases per group in this trial.

### 2.7. Statistical Analyses

Statistical Package for Social Sciences (SPSS) software Version 21.0 was used for statistical analysis. The normality of continuous data was tested using the Shapiro–Wilk test. Continuous data were presented as the mean ± standard deviation (SD) or median (interquartile range [IQR]). Independent Student's *t*-test was used for normally distributed variables, and the Mann–Whitney *U* test was used for the nonparametric variables. Differences in the continuous data of the PI, PI ratio, MAP, and HR were tested by applying repeated measures of ANOVA with post hoc pairwise comparisons using the Bonferroni test. Categorical variables were described as numbers (percentages) and analyzed by the Chi-square test or Fisher's exact test. A receiver operating characteristic (ROC) curve was established to evaluate the effect of PI at 10 min and PACU predicting the block. The best cutoff value was obtained when the Youden index was maximum. *p* value < 0.05 was considered statistically significant.

## 3. Results

**Figure 1:**
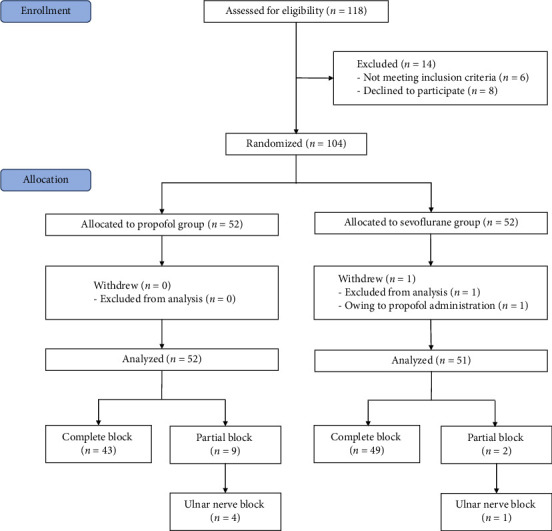
Flow diagram of included participants.

In total, 118 children were screened for eligibility, and 104 subjects were enrolled and randomly assigned to either the PRO group or the SEV group (Figure 1). One case that changed the anesthesia regimen was excluded, and 103 patients were available for the final analysis. The block was successful in 43 (82.69%) and 49 (96.08%) patients in the PRO group and SEV group, respectively. Participant characteristics between the PRO group and the SEV group were comparable (Table 1).

### 3.1. Comparison of the PRO Group and SEV Group

**Figure 2:**
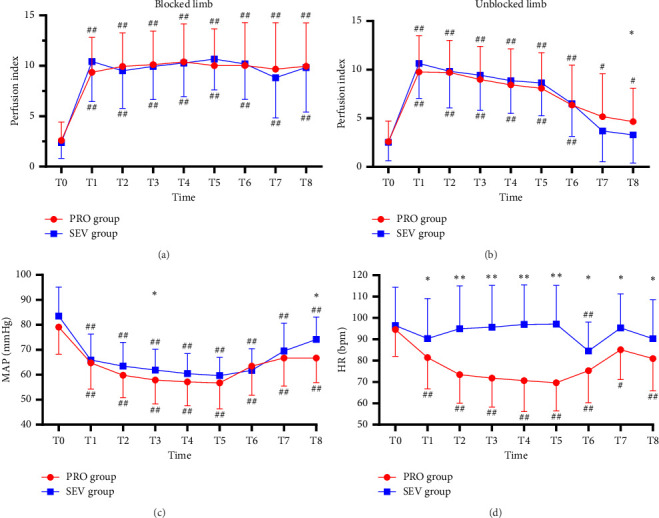
Change trends of the PI, MAP, and HR in the PRO group (*n* = 43) and the SEV group (*n* = 49). (a) PI in the blocked limb, (b) PI in the unblocked limb, (c) MAP, and (d) HR. Before the anesthesia induction (T0, baseline), immediately after LMA placement (T1), 0 min (T2), 5 min (T3), 10 min (T4), 15 min (T5) after SCB, immediately after surgery (T6), after removal of LMA (T7), and in PACU (T8). ⁣^∗^*p* < 0.05 and ⁣^∗∗^*p* < 0.001 compared between two groups, ^#^*p* < 0.05 and ^##^*p* < 0.001 compared with baseline.

For the blocked limb, PI was comparable at all time points in both groups (all *p* > 0.05) (Figure 2(a)). For the unblocked limb, the PI of the index finger in PRO group was higher than that in the SEV group at T8 (*p*=0.042), while there was no significant difference in PI between the two groups at other time points (all *p* > 0.05) (Figure 2(b)). Besides, MAP exhibited no significant difference between the two groups (all *p* > 0.05), except T3 (*p*=0.034) and T8 (*p* < 0.001), at which MAP in the SEV group was higher than that in the PRO group (*p*=0.042) (Figure 2(c)). HR in the PRO group was significantly lower than that in the SEV group at T1–T8 (all *p* < 0.05) (Figure 2(d)).

### 3.2. Comparison With Baseline

On the blocked side, PI increased significantly at T1 compared with the baseline (*p* < 0.001) and then remained stable from T1 to T8 (Figure 2(a)). Similarly, for the unblocked side, the index finger PI in the PRO group at T1–T8 was higher than that at T0 (*p* < 0.001) (Figure 2(b)). While PI in the SEV group was higher at T1–T6 compared with that at T0 (*p* < 0.001) but had no significant difference at T7–T8 (*p* > 0.05). In addition, MAP in both groups declined rapidly after anesthesia induction and remained smaller than T0 despite an upward trend after the end of anesthesia (all *p* < 0.001). HR in the PRO group decreased significantly following anesthesia induction compared with T0 (all *p* < 0.05), while it depicted smaller fluctuations in the SEV group except T6 (*p* < 0.001) (Figure 2(d)).

### 3.3. Comparison of Blocked and Unblocked Limbs

**Figure 3:**
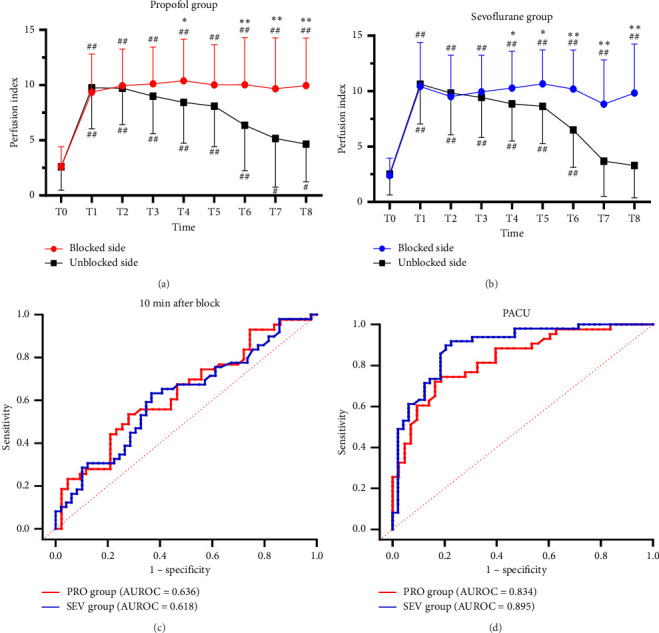
Change trends of the PI after successful block. (a) PI in the PRO group (*n* = 43), (b) PI in the SEV group (*n* = 49), (c) the ROC curve of the PI at 10 min, and (d) the ROC curve of the PI at PACU. Before the anaesthesia induction (T0, baseline), immediately after LMA placement (T1), 0 min (T2), 5 min (T3), 10 min (T4), 15 min (T5) after SCB, immediately after surgery (T6), after removal of LMA (T7), and in PACU (T8). ⁣^∗^*p* < 0.05 and ⁣^∗∗^*p* < 0.001 compared between two groups, ^#^*p* < 0.05 and ^##^*p* < 0.001 compared with baseline.

For complete block, the PI at T0–T3 was comparable between blocked and unblocked limbs in both groups (all *p* > 0.05) (Figures 3(a) and 3(b)). From T4 to T8, PI of the blocked side was higher than that of the unblocked side (all *p* < 0.05). In addition, there was no significant difference in the PI ratio on the blocked side compared to that on the unblocked side in both the PRO group (6.2 [4.8] versus 6.2 [6.5]; *p* > 0.05) and the SEV group (8.2 [8.2] versus 5.9 [5.2]; *p* > 0.05).

### 3.4. The Predictive Effect of PI

ROC curves were constructed based on PI in the blocked and unblocked limbs (Figures 3(c) and 3(d)). At 10 min after block, AUROCs were 0.636 (95% CI 0.519–0.753) and 0.618 (95% CI 0.506–0.729), respectively, in the PRO group and the SEV group (Table 2). The cutoff values for the effect of SCB under propofol and sevoflurane anesthesia were 8.51 and 9.48, respectively, with low sensitivity and specificity. However, at PACU, PI showed a high sensitivity (0.837 vs. 0.796) and specificity (0.721 vs. 0.898) for SCB at cutoff values of 5.91 and 6.67 in both the PRO group and the SEV group. The AUROC curves were 0.834 (95% CI 0.750–0.918) and 0.895 (95% CI 0.832–0.959), respectively.

### 3.5. Other Outcomes

The anesthesia time, operative time, PACU stay time, hospital stays, agitation scores, and pain scores showed no significant differences between the two groups (all *p* > 0.05) (Table 3). However, the PRO group exhibited a shorter LMA removal time compared to the SEV group (*p* < 0.001). Additionally, there were no cases of pneumothorax, nerve damage, and local anesthetic intoxication, and the incidence of nausea and vomiting (1.92% vs. 1.96%, *p* > 0.999), pruritus (0 vs. 5.88%, *p*=0.118), and fever (5.77% vs. 1.96%, *p*=0.618) was similar in both groups. In the failure cases, the supplemental remifentanil dose averaged 320.72 ± 176.03 μg in the PRO group and 259.20 ± 223.16 μg in the sevoflurane group, which had no statistically significant difference observed between the two groups (*p*=0.675).

## 4. Discussion

PI is considered to be an objective, rapid, and efficient indicator in predicting the success of regional anesthesia when administered to awake patients [5, 6]. In this study, we observed the variations of PI in children scheduled for SCB under propofol or sevoflurane anesthesia and found that at 10 min after SCB, the PI of the blocked side was significantly higher than that of the unblocked side, but the sensitivity and specificity in predicting the effect of block were low. However, the PI measured during the anesthesia recovery period had high sensitivity and specificity in predicting the effective block and was expected to be an indicator to guide postoperative supplemental analgesia.

Changes in PI under GA are complex due to the influence of anesthetics, pain, volume status, and others [6]. This study found that the trend of PI for the unblocked side was mainly divided into three phases according to GA. The initial rapid rise reflects vasodilation following the administration of anesthetic induction agents. Subsequently, the slow decline to gradual stabilization represents a shift in vasodilation from the loading dose to the maintenance dose. The final downslope of the trendline reflects the disappearance of vasodilation owing to the metabolism of anesthetics and the sympathetic excitation during recovery [16, 17]. The trendline for PI in the blocked limb showed an upward-steady pattern. Similar to the unblocked side, PI experiences a rapid increase in the first phase. In the second phase, PI showed relative stability, which may be attributed to the fact that the vasodilation induced by the initial anesthetic loading dose had reached its maximum effect, while the sympathetic block facilitated its maintenance at this level. Sebastiani et al. proposed that the difference of PI between the blocked and unblocked sides followed by SCB disappeared after anesthesia induction, possibly due to vessels being maximally dilated, which supports our findings [18].

Recently, studies have been reported on the predictive effect of PI on SCB administered under GA in adults. Shah et al. performed SCB to oncoorthopedic patients aged 15–80 years after the completion of surgery and before the end of anesthesia [17]. They found that PI increased within 5 min after block, while PI of the opposite side failed to increase. Ceylan and Eşkin showed that the PI change rate significantly increased in the 5^th^, 10^th^, and 20^th^ min compared to baseline in adult patients under propofol- and sevoflurane-combined anesthesia [19]. In this study, we observed that PI in the blocked limb was higher than that in the unblocked side at 10 min after block, and this difference persisted into PACU. This is similar to previous research [17, 19]. Different from other studies, the participants of this study were children under GA, and the anesthesia protocol was either propofol intravenous administration or sevoflurane inhalation, and the implementation time of SCB was after anesthesia induction.

However, the cutoff value of PI at 10 min has low sensitivity and specificity in predicting effective block, especially compared to SCB administered awake [7, 8, 20]. During the recovery period of GA, PI showed a well-predictive effect, which may be attributed to the decrease of PI in the unblocked side. Thus, PI measured during the recovery period is a superior predictor for SCB to guide postoperative analgesia. Ahmed et al. observed that PI was negatively correlated with pain score at 30 and 90 min postoperatively in children who underwent adenotonsillectomy and suggested that PI is a good objective indicator to predict the presence of postoperative pain [21]. In our study, due to the high rate of success block, the median pain score of children in PACU was 0. Future studies can focus on the predictive effect of PI on postoperative pain in failed blocks.

In this study, none of the patients experienced block failure. The variation of PI in nine patients with a partial block showed a similar trend to that of patients with complete block in the PRO group, suggesting that PI seemed to have difficulty distinguishing partial block from complete block under propofol anesthesia. In the study of Abdelhamid et al., the PI ratio of the index finger increased after block and did not reflect ulnar nerve sparing [22]. They demonstrated that the increase in PI after SCB is segment-dependent. Therefore, the PI value of the index finger does not reflect all types of partial blocks, especially under GA when peripheral blood vessels have been dilated.

Propofol can effectively inhibit myocardial contractility and sympathetic nerve activity, followed by significant dose-dependent vasodilation [23–25]. Ma et al. reported that 1%–3% sevoflurane inhibits somatic sympathetic nerve reflexes and causes a reduction in systemic vascular resistance [26]. Since PI can reflect changes in peripheral vascular resistance, some studies have used it as a quantitative surrogate for drug-induced vasoconstriction or vasodilation [10, 27]. In this study, we observed a similar variation of PI following propofol or sevoflurane anesthesia, suggesting that they exert similar vasodilator effects. However, during the recovery period from anesthesia, the SEV group had a faster decline in PI than the PRO group and showed a lower PI in PACU. Similarly, after anesthesia reversal, MAP in the SEV group had faster recovery than in the PRO group. Those results indicated that sevoflurane showed a faster recovery in systemic vascular resistance and MAP compared with propofol [25]. Additionally, sevoflurane anesthesia may potentiate the effects of peripheral nerve blocks owing to its skeletal muscle relaxant properties, a factor not present with propofol [28, 29]. However, it still lacks direct comparisons between the two anesthetics on the efficacy of nerve block and future studies can explore this area further.

Our study protocol underwent a thorough peer review process (https://trialsjournal.biomedcentral.com/articles/10.1186/s13063-022-06597-y) [30]. There were still some limitations. Firstly, because ultrasound guidance increases the probability of complete block, it is difficult to calculate the correlation between PI of little fingers and ulnar sparing. Future studies can further expand the sample size to obtain a sufficient number of cases of ulnar sparing. Secondly, our observations were limited to a 15-min period following SCB, which may have resulted in the omission of certain outcomes, such as delayed block. Third, the inhaled anesthetic sevoflurane has skeletal muscle relaxation properties, particularly exhibiting a synergistic effect with muscle relaxants, which may affect the assessment of the block. Nonetheless, as the patient recovers from anesthesia, the effects of sevoflurane diminish over time.

## 5. Conclusion

PI exhibits limited sensitivity and specificity in predicting the effect of SCB at 10 min after block under GA, irrespective of the anesthetic agent used, whether propofol or sevoflurane, while PI exhibited high sensitivity and specificity for SCB at cutoff values of 5.91 and 6.67 both after propofol or sevoflurane anesthesia during PACU, which may be beneficial to guide postoperative supplementary analgesia according to the effect of block.

## Figures and Tables

**Table 1:** Characteristics of patients.

	PRO group (*n* = 52)	SEV group (*n* = 51)	*p* value
Age (yr)	6.5 (5.0–10.0)	7.0 (5.0–9.0)	0.703
Sex			0.391
Male	34 (65.4%)	38 (74.5%)	
Female	18 (34.6%)	13 (25.5%)	
Height (cm)	128.1 ± 21.9	127.0 ± 18.5	0.781
Weight (kg)	24.2 (18.7–37.3)	26.4 (20.0–31.0)	0.913
BMI (kg·m^−2^)	15.7 (14.4–18.1)	15.7 (14.4–17.5)	0.654
Hemoglobin (g·dl^−1^)	12.5 ± 0.9	12.3 ± 1.1	0.433
Type of surgery			0.386
Ulnar and radial fractures	23 (44.2%)	20 (39.2%)	
Humerus fracture	20 (38.5%)	21 (41.2%)	
Elbow deformity	2 (3.8%)	6 (11.6%)	
Others	7 (13.5%)	4 (7.8%)	

*Note:* Data were expressed by mean ± SD, median (IQR), or frequencies (percentages).

Abbreviations: ASA, American Society of Anesthesiologists; BMI, body mass index.

**Table 2:** ROC for the ability of PI to predict the effectiveness of block.

Group	Time	AUROC (95% CI)	Sensitivity (%)	Specificity (%)	Cutoff value
PRO	10 min	0.636 (0.519–0.753)	0.721	0.535	8.51
	PACU	0.834 (0.75–0.918)	0.837	0.721	5.91

SEV	10 min	0.618 (0.506–0.729)	0.633	0.633	9.48
	PACU	0.895 (0.832–0.959)	0.796	0.898	6.67

Abbreviations: AUROC, area under the receiver operating characteristics; PI, perfusion index; PRO, propofol; ROC, receiver operating characteristics; SEV, sevoflurane.

**Table 3:** Comparison of clinical data between propofol and sevoflurane groups.

	PRO group (*n* = 52)	SEV group (*n* = 51)	*p* value
Anesthesia time (min)	121.5 (102.5–144.8)	121.0 (100.0–143.0)	0.856
Surgery time (min)	81.0 (60.5–104.8)	79.0 (54.0–104.0)	0.386
LMA removal time (min)	7.0 (5.0–10.0)	10 (7.0–13.0)	< 0.001
PACU stay time (min)	27.5 (18.3–40.0)	30 (22.0–38.0)	0.843
Hospital stays (day)	7.0 (5.0–8.0)	7.0 (5.0–8.0)	0.665
Watcha score	1.0 (1.0–2.0)	1.0 (1.0–1.0)	0.250
CHIPPS score			
PACU	0 (0–0)	0 (0–0)	0.293
PD1	0 (0–1)	0 (0–2)	0.683
PD2	0 (0–0)	0 (0–0)	0.738
Diclofenac sodium suppositories (mg)	0 (0–10.9)	0 (0–12.5)	0.075

*Note:* Data were expressed by median (IQR).

Abbreviations: CHIPPS, the Children's and Infants' Postoperative Pain Scale; LMA, laryngeal mask; PACU, postanesthesia care unit; PD, postoperative day.

## Data Availability

The data that support the findings of this study are available from the corresponding author upon reasonable request.
